# Building the next generation of family medicine and primary health care researchers in Africa

**DOI:** 10.4102/phcfm.v17i2.5274

**Published:** 2025-12-05

**Authors:** Robert J. Mash, Klaus von Pressentin

**Affiliations:** 1 Division of Family Medicine and Primary Care, Faculty of Medicine and Health Sciences, Stellenbosch University, Cape Town, South Africa; 2 Division of Family Medicine, Department of Family, Community and Emergency Care, University of Cape Town, Cape Town, South Africa

The World Health Organization (WHO) identified research as one of the key operational levers for implementing primary health care (PHC).^1^ They advocate for developing research networks, performing implementation research to scale up effective interventions and involving communities in developing a shared research agenda. However, the contribution of Africa to global health science is between 1% and 2% despite the vast size, population and health needs of the continent.^2^ The contribution of PHC research is likely even less.

A recent review of family practice research in Africa concluded that we need to move away from small-scale descriptive research to larger, collaborative and interdisciplinary studies that can tackle priority research questions.^3^ We need more research outside of South Africa, more focus on comprehensive care (especially children, palliative and rehabilitative care), as well as continuity and coordination. Research should also consider PHC as a ‘whole of society’ approach,^4^ and not just clinical care. This would mean addressing multisectoral action and community empowerment. To provide health systems, services and communities with the critical evidence, we need to develop more researchers.^5^

**FIGURE 1:**
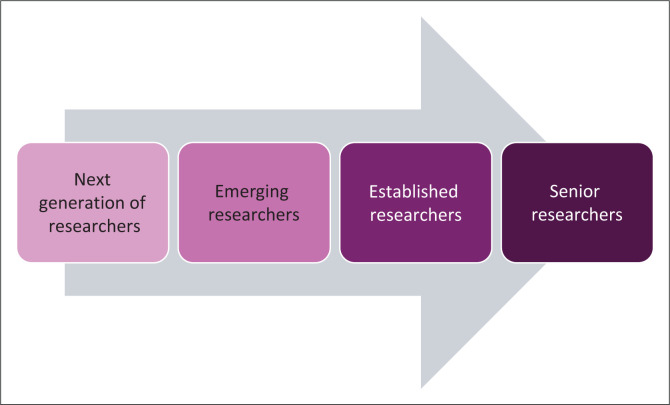
The research capacity building pipeline.

Developing an established researcher requires explicit and dedicated attention to all stages of the capacity-building pipeline (Figure 1). The next generation of researchers usually begins their journey with a master’s degree or, in some countries, a fellowship qualification. For most, this marks the end of their engagement with research, higher education, as their focus shifts to becoming a specialist clinician or consultant. From this pool, only a few go on to complete a doctoral degree, and although this is regarded as the most advanced degree you can obtain from a university, it is just the beginning of a longer journey towards becoming an established or senior researcher.

Once the next generation of researchers have identified themselves through obtaining a doctoral degree or by just building a body of work, they emerge as future researchers. In some disciplines, researchers will develop further as postdoctoral fellows; however, in clinical disciplines, these positions do not usually make financial or career sense. Emerging researchers usually need to have an active mentor, often an academic appointment, and to build on the foundation of their doctoral work by conducting further studies. As they gain experience, they should build international collaborations, successfully apply for international grants and be rated as researchers by their national bodies. At this point they have become established researchers and often supervise several postgraduate students. For some established researchers, they may have the opportunity to develop larger strategic initiatives, centres of excellence or to occupy research chairs. At this point they become senior researchers who manage larger research endeavours, departments or institutions. It is vital to ensure equitable access to research infrastructure and resources at institutional, national and international levels to provide stage-specific support and funding for capacity building.^6^

One can regard the acquisition of a doctoral degree as a critical step in the pipeline when the next generation of researchers step forward with the intention of becoming established researchers and leaders in their discipline. In 2025, the *African Journal of Primary Health Care and Family Medicine* published a special collection of articles aimed at supporting doctoral researchers. This series builds on the primary care research methods series from 2014 that was aimed more at master’s level students. The new series has 11 articles that cover a range of more advanced topics:

- *Writing for a Doctor of Philosophy (PhD): A guide to developing a strong and coherent thesis*.^7^
- *From dreamers to doers: Navigating the doctoral journey in family medicine and primary care*.^8^
- *Phenomenology for primary care researchers*.^9^
- *Understanding implementation research*.^10^
- *Clinical trial methods for family medicine and primary care*.^11^
- *Cost-effectiveness analysis in primary care research: A practical guide for early-career researchers*.^12^
- *Integrating evidence synthesis into doctoral research: A guide for family medicine and primary care*.^13^
- *From separate streams to confluence: A framework for meaningful mixed methods integration in African primary care research*.^14^
- *Design science research in quality improvement: Embedding rigour in digital health innovation*.^15^
- *Real-world evidence for primary care: A primer on observational research*.^16^
- *Integrated knowledge translation: A guide for primary care researchers*.^17^

We hope that these articles will support doctoral researchers in developing their research proposals. The development of a research question, an initial concept note and a full proposal takes time, often a year, and requires assistance. A cohort model may help a group of prospective doctoral students to think together with the support of more experienced mentors or supervisors.^18^

It is important to focus the available supervisory capacity on assisting people who have the intention of becoming established researchers. The number of established researchers who have supervised doctoral students to completion is so small that we must make the most of this scarce resource to support the pipeline and grow capacity. The supervisors should carefully select the students they agree to supervise. In some contexts, collaboration with supervisors from high-income and better-resourced settings or related disciplines has helped more doctoral students progress.^19^

Clinician-scholars require specific strategies to complete their doctoral degrees. Doing a PhD part-time by publication rather than full-time or by traditional thesis can make the process more manageable and feasible. Having funding to buy out dedicated time at key moments, such as data collection and analysis stages, can help advance the project. However, a lack of funding may require doctoral researchers to maintain full-time employment.^20^ In some settings, a cohort model of supervision may also be helpful, especially by creating a community of practice and opportunities for shared learning and support.^21^

The number of emerging researchers with a doctoral degree in the field of family medicine and primary care is quite limited in sub-Saharan Africa. Unfortunately, these emerging researchers often have many other clinical and educational duties, which sometimes take precedence. One cannot assume that an emerging researcher with a PhD will immediately be a competent supervisor. On the one hand, it is important for potential supervisors to start immediately, but on the other hand, they will need training and mentoring for this role. More established or senior researchers should co-supervise with the intention of building supervisory capacity. There are also available training courses on postgraduate supervision, such as the African Doctoral Academy.^22^

The primary care research methods special collection is, therefore, a valuable resource for both doctoral researchers and their novice supervisors. We hope that it will help expand the pipeline of emerging and established researchers in the fields of family medicine and primary health care.
